# Resistance training improves metainflammation and body composition in obese adolescents

**DOI:** 10.1186/1687-9856-2015-S1-O40

**Published:** 2015-04-28

**Authors:** Rachana Dahiya, Sarah Shultz, John Cardinal, Nuala Byrne, Andrew Hills, Kristen Gibbons, Louise Conwell, Mark Harris, Gary Leong

**Affiliations:** 1 School of Medicine, The University of Queensland, Herston, QLD, Australia; 2 School of Sport & Exercise, College of Health, Massey University, Wellington, New Zealand; 3 Pathology Queensland, Herston, Australia; 4 Institute of Health and Biomedical Innovation, Queensland University of Technology, Brisbane, QLD, Australia; 5 Mater Research - UQ, South Brisbane, QLD, Australia; 6 Royal Children's Hospital, Brisbane, QLD, Australia; 7 Mater Children's Hosp, South Brisbane, QLD, Australia; 8 The University of Queensland and Mater Children's Hospital, St Lucia, QLD, Australia

## 

Adolescent obesity is associated with inflammation, insulin resistance and prediabetes. The purpose of the study was to investigate whether resistance training (RT) improves the adipokine profile, body composition and insulin sensitivity of obese adolescents. Fourteen obese adolescents (16.1+1.6 y; M:F 6:8; body mass index (BMI) SDS 2.01 + 0.31) were recruited for a 16-week RT intervention. Thirty-one lean youth (15.6+1.3 y; M:F 19:12; BMI SDS -0.03+0.07) had baseline anthropometric measurements and blood tests for comparison. Participants completed 3 RT sessions per week with training load progressively increased from 60% to 85% of one repetition maximum (1-RM). The following parameters were examined pre- and post-intervention: 1) Height, weight, BMI; 2) High sensitivity C-reactive protein (hs-CRP) and adipokines including interleukin (IL)-1β, IL-6, tumor necrosis factor-alpha (TNF-α), adiponectin, soluble intercellular adhesion molecule (sICAM)-1, leptin and resistin; 3) Body composition by dual energy x-ray absorptiometry (DXA), and 4) Insulin sensitivity by homeostatic model assessment (HOMA-IR). Obese youth had significantly higher IL-1β, leptin and resistin (all p<0.0001) and lower adiponectin and sICAM-1 (both p<0.0001) at baseline compared with lean youth. Post-intervention, a reduction in IL-6 (p<0.01), IL-1β (p<0.01) and resistin (p <0.001) was observed whereas adiponectin and sICAM-1 increased (p<0.05, p<0.001 respectively) in obese adolescents. HOMA and hs-CRP remained unchanged. IL-1 β and sICAM-1 levels in obese youth normalised to levels comparable to lean youth post-intervention (p = 0.1 and p=0.2), whereas TNF-α and resistin were significantly lower (p<0.05, p<0.01 respectively) post-intervention in the obese youth. Percent fat for the trunk, arm and total body (p<0.05) were reduced, while fat free mass (p<0.01) was increased after the intervention. RT is a feasible intervention to improve the adipokine profile and body composition of obese adolescents suggesting it may be a feasible intervention for improving metabolic health in obese youth at high risk of Type 2 Diabetes.

